# Evidence of reduced birthweight in Ukraine following the Russian invasion

**DOI:** 10.1038/s41598-025-98668-8

**Published:** 2025-04-25

**Authors:** Svitlana Arbuzova, Margaryta Nikolenko, Liubov Atramentova, Howard Cuckle

**Affiliations:** 1 Eastern-Ukrainian Center for Medical Genetics and Prenatal Diagnosis, Mariupol & Kyiv, Ukraine; 2 Institute of Health Research, University of Exeter, 16 Trinity Apartments, 3 Roman Wall, Exeter, Devon, EX1 1GP England, UK; 3 Department of Genetics and Cytology, Kharkiv National University Named V.N. Karazin, Kharkiv, Ukraine; 4 Faculty of Medicine, Tel Aviv University, Tel Aviv, Israel

**Keywords:** Screening, Prenatal, War, Birth-weight, Weight length ratio, Health care, Medical research

## Abstract

The impact of armed conflict on birth weight has been shown in various studies, but the effects of the full-scale Russian invasion of Ukraine in 2022 have been overlooked until now. This study investigates the influence of war on birth-weight using data and follow-up of pregnancies among women having prenatal screening. Maternal factors, outcomes, and birth weights were analyzed for 706 screened women who delivered between 2020 and 2023, excluding cases of spontaneous abortion, termination, and multiple pregnancies. Of these, 330 deliveries occurred before the invasion and 376 afterward. The only statistically significant maternal factors were parity − 22% nulliparous before and 48% after invasion (*P* < 0.0001) and maternal weight − 60 versus 62 kg. (*P* < 0.05). Birth-weight was significantly reduced: median 3500 g before and 3350 g after (*P* < 0.0001); proportions < 2500 g, 1.2% and 2.4%, respectively (*P* = 0.24). The ratio of birth-weight to infant length was also significant (*P* < 0.0001). Analysis of variance showed birth-weight significances remained after allowing for parity and gender. These findings highlight a significant decline in birth weight associated with armed conflict, emphasizing the need for targeted interventions to support maternal and neonatal health during periods of war.

## Introduction

Several studies have shown that armed conflict can have a detrimental effect on pregnancy outcome. A systematic review on the subject included 9 studies in women from Iraq, Kuwait, Libya, Croatia, Bosnia and Herzegovina, Somalia, Kosovo and Afghanistan, all reporting a significant association with low birth-weight (< 2500 g)^1^. A subsequent study in Colombia found a 3–4 fold increase in very low birth-weight (< 1500 g) among exposed pregnancies^2^. Another review combined data from 53 developing countries for the period 1990–2018, including more than 294,000 births^3^. The average birth-weight in exposed pregnancies was 3104 g and in exposed pregnancies it was 3017 g. The corresponding proportions with low birth-weight were 11.2% and 13.3% respectively.

Ukraine has been subjected to localized armed conflict since 2014 and national warfare with the full-scale invasion of the country by Russia in February 2022. This prompted us to investigate birth-weight changes in Ukraine before and after the invasion, using a database of women having prenatal screening tests.

We hypothesize that the invasion will have reduced birth-weight, on average, and increase the rate of low birth-weight pregnancies. A number of potential factors might contribute to such an outcome including: overburdened health-care provision, displacement, food insecurity, and traumatic stress.

## Methods

Women who had a first trimester Combined screening test for Down syndrome at the Kyiv branch of the Eastern-Ukrainian Center for Medical Genetics and Prenatal Diagnosis were followed-up to determine the outcome of pregnancy. The test is provided free of charge under a National Health Service of Ukraine package entitled ‘Outpatient Pregnancy Management’. Staff at the centre are certified to perform screening procedures by the Fetal Medicine Foundation (FMF) and carry out international level research according to FMF standards.

All women attending for screening were eligible for the study except those likely to have a high risk pregnancy. The exclusion criteria were: antiphospolipid syndrome and, in multiparous women, a previous history of preeclampsia or intra-uterine growth restriction.

Information on several factors was routinely obtained at the time of presentation for screening and stored in the Astraia prenatal database (Astraia Gmbh, Munich, Germany). These comprised gestational age, maternal age, parity, mode of conception, maternal weight and height. Data on birth-weight, fetal length, gestation of delivery and mode of delivery were obtained from a questionnaire sent to each patient. If not returned or incomplete, this was followed by telephone contact. Only those with complete information were included in the study. Pregnancies that ended in spontaneous abortion or elective termination were excluded.

The principle endpoints were birth-weight and fetal length. The ratio between weight and length was also computed as a marker of fetal nutritional status^4^. In addition, follow-up information was collected on gestation of delivery, mode of delivery and gender. Informed consent was obtained from all individual participants included in the study.

### Statistics

For all information collected at the time of screening and delivery, statistical significance between those before and after the Russian invasion was assessed by the Wilcoxon Rank Sum Test for continuous variables and the Chi-square Test for categorical variables. For all variables, a 2-tail test was performed.

For birth-weight, separate comparisons were carried out on male and female fetuses, as well as analysis of variance (ANOVA) to allow for gender. To assess any confounding variables at the time of screening found to be statistically significant were also entered into an ANOVA; in a multi-variate analysis all variables at the time of screening were used.

The study design assumes that the exposure to the affects of the Russian invasion among those attending for prenatal screening is representative of all pregnant women in the Ukraine. Ideally, information on exposure to risks related to the war would be available in screened women and, for comparison, a series of unscreened women. The statistical approach would then be to perform a difference-in-difference analysis. However, it was not feasible to collect this information in screened and unscreened women. We therefore rely on the simpler statistic of an ANOVA to control for any variables that might be associated with birth-weight and possibly exposure.

All computations were carried out using SAS software (version 9.4; SAS Institute Inc., Cary, North Carolina).

### Ethical approval

All procedures and methods were performed in accordance with the Declaration of Helsinki and the study’s protocol received approval from the Ethics Committee of Eastern-Ukrainian Center for Medical Genetics and Prenatal Diagnosis, Ukraine (148/3/12/24). The women gave informed consent and signed informed consent forms.

## Results

### Participants

A total of 706 women screened for Down syndrome at the Eastern-Ukrainian Center for Medical Genetics and Prenatal Diagnosis in 2020-23 and delivered before December 2023 were included in the study. Of these 330 were screened and delivered before 24 February 2022, the date of the Russian invasion, and 376 afterwards. All screening tests were carried out at 11–13 weeks of gestation and women included in the study had prenatal care throughout pregnancy.

### Comparative analysis of baseline characteristics

Table 1 shows that there was no statistically significant difference between the time periods in gestational age, maternal age, mode of conception and maternal height. However, there was a highly statistically significant difference in parity with an approximately 2-fold higher proportion of nulliparous women, in those screened after the Russian invasion (*P* < 0.0001). Maternal weight was slightly increased as was body mass index (weight/height^2^).

**Table 1:** Maternal characteristics before and after the invasion.

Characteristic	Before (*n* = 330)	After (*n* = 376)	*P*-value*
Gestation (day)			
Median	87	88	0.40
IQR	85–90	85–90	
Maternal age			
Median	31	31	0.70
IQR	28–34	27–35	
≥ 35	24% (80/330)	27% (102/376)	0.38
Parity			
Nulliparous	22% (72/330)	48% (182/376)	< 0.0001
Multiparous	78% (258/330)	52% (194/376)	
Conception			
ART	3% (9/330)	3% (12/376)	0.72
Spontaneous	97% (321/330)	97% (364/376)	
Weight (kg)			
Median	60	62	< 0.05
IQR	54–68	56–70	
Height (cm)			
Median	166	165	0.29
IQR	163–170	162–170	
BMI (kg/m^2^)			
Median	21.8	22.5	< 0.005
IQR	20.2–24.1	20.5–25.5	
≥ 25	19% (64/330)	28% (105/376)	< 0.01

IQR = inter-quartile range; ART = assisted reproduction technology; BMI = body mass index.

*Median & IQR from Wilcoxon Rank Sum Test, proportion from Chi-square Test, both 2-tail.

Table 2 shows that there was no statistically significant difference between the time periods in gestational age at birth, gender or mode of delivery. However, birth-weight was highly significantly reduced, with medians of 3500 g before and 3350 g after the invasion (*P* < 0.0001). The rate of low birth-weight pregnancies was greater after invasion than before − 1.2% and 2.4%, respectively - although this did not reach statistical significance (*P* = 0.24). Infant length was also slightly reduced (*P* < 0.005) and the birth-weight length ratio remained highly significant (*P* < 0.0001).

**Table 2:** Delivery factors before and after the invasion.

	Before (*n* = 330)	After (*n* = 376)	*P*-value*
Gestation (wk)			
Median	40	40	0.54
IQR	39–40	39–40	
Gender			
Male	52% (172/330)	54% (210/376)	0.67
Female	48% (158/330)	46% (174/376)	
Delivery			
C-section	24% (78/330)	20% (76/376)	0.27
Not	76% (252/330)	80% (300/376)	
Weight (g)			
Median	3500	3350	< 0.0001
IQR	3240–3800	3100–3600	
< 2500 gm	1.2% (4/330)	2.4% (9/376)	0.24
Length (cm)			
Median	53	52	< 0.005
IQR	52–55	51–54	
Ratio (kg/m)			
Median	6.54	6.37	< 0.0001
IQR	6.15–6.98	6.06–6.73	

IQR = inter-quartile range.

*Median & IQR from Wilcoxon Rank Sum Test, proportion from Chi-square Test, both 2-tail.

Table 3 shows that the magnitude of difference in median birth-weight and birth-weight/infant length between the pre-and post-war periods were greater for males than females, and these differences only reached statistical significance in males. ANOVA showed that the difference in birth-weight was significant after allowing for gender (F = 12.3, *P* < 0.0001) and there was no significant interaction between gender and period (F = 3.8, *P* = 0.05). The corresponding values for the ratio between birth-weight and infant length were F = 14.1, *P* < 0.0005 and F = 2.5, *P* = 0.11, respectively.

**Table 3:** Birth-weight before and after the invasion, according to gender.

	Before	After	*P*-value*
Male			
Number	172	202	
Median weight (g)	3600	3370	< 0.0001
IQR	3310–3875	3140–3600	
Median length (cm)	54	53	< 0.0005
IQR	52–56	51–54	
Median ratio (kg/m)	6.62	6.37	< 0.0001
IQR	6.27–7.09	6.08–6.67	
Female			
Number	158	174	
Median weight (g)	3415	3330	0.10
IQR	3180–3700	3100–3600	
Median length (cm)	52	52	0.56
IQR	51–54	51–54	
Median ratio (kg/m)	6.46	6.36	0.10
IQR	6.13–6.86	6.02–6.77	

IQR = inter-quartile range.

*Median & IQR from Wilcoxon Rank Sum Test, proportion from Chi-square Test, both 2-tail.

The results on birth-weight are confounded by parity since weight is lower in nulliparous pregnancies^5^. However, ANOVA showed that the difference in birth-weight between the time periods was significant after allowing for nulliparity (F = 19.3, *P* < 0.0001) and the difference in birth-weight between nulliparous and multiparous women not was significant after allowing for period (F = 0.4, *P* = 0.52). The corresponding values for the ratio between birth-weight and infant length were F = 14.1, *P* < 0.0005 and F = 1.7, *P* = 0.20, respectively.

Multi-variate ANOVA using all six maternal variable, whether statistically significant or not (Table 1), showed that the difference in birth-weight between the time periods remained significant (F = 19.2, *P* < 0.0001), as did the ratio between birth-weight and infant length (F = 14.3, *P* < 0.0002).

An additional series of 37 pregnancies was identified among women who attended the Kyiv branch of the Eastern-Ukrainian Center for Medical Genetics and Prenatal Diagnosis after the Russian invasion. All of them attended after 13 weeks gestation, too late for the Down syndrome screening Combined test. The median birth-weight was 3200 g, statistically significantly lower than the median of 3350 g in women attending before 13 weeks post-war (*P* < 0.05). This reinforces the finding of reduced birth-weight associated with the war but begs the question why late attendance would be related to birth-weight. Socio-economic status (SES) might be an explanation but such data is not routinely collected at the screening centre and this variable cannot be evaluated in the current study.

## Discussion

We have demonstrated a reduction in birth-weight following the full-scale Russian invasion of Ukraine. This was found in a series of women attending for first trimester Down syndrome screening in Kyiv.

The median birth-weight in the post-war period was 150 g (4.3%) lower than the median birth-weight before the war. Whilst this is only a moderate change, on average, it represents a general downward shift in the whole distribution of weights, as can be seen from the change in the inter-quartile range (see Table 2). Hence, the number of newborns at risk for complications of pregnancy will have increased with potential detrimental development and life-long health consequences^6^.

There is no reason to believe that the effect of the war on birth-weight would differ between screened and unscreened women. Moreover, the fact that screening was carried out in the first trimester is advantageous, since women attending after the invasion would have been exposed to potential hazards throughout most of their pregnancy.

However, there is evidence from other countries that women attending for prenatal screening have a higher SES than non-attendees. A study in the Netherlands, which generally has a low uptake of the Combined screening test and requires co-pay unless advanced maternal age, found a 17% uptake in women of below median SES and 27% in those above median SES^7^. A survey in the UK, where prenatal screening for Down syndrome is free of charge - like in Ukraine - found a 63% uptake for women living in postcodes above the upper quintile Index of Multiple Deprivation (IMD) compared with 71% in the remainder^8^. It is also possible that women of higher SES and lower IMD would have the resources to ameliorate the impact of war on birth-weight.

Expanding the number of pregnancies included in the study became increasingly difficult due to emigration and changing contact details after the invasion. This phenomenon should be borne in mind when planning studies like ours. Patterns of migration are likely to differ according to nature of the conflict, geographical factors and any restrictions to leaving a combat zone or entering another country. The individual circumstances may also influence the health outcome being measured.

A possible confounding maternal factor was parity. The nulliparous rate was almost two times higher after the invasion than before, and nulliparity is associated with lower birth-weight^5^. There was no change in screening policy over this period and the only likely explanation is that more nulliparous women did not provide follow-up information after the war. Whatever the cause, ANOVA showed that the reduction in birth-weight remained statistically significant after allowing for parity.

This study represents the first systematic investigation of birth-weight changes before and after the full-scale Russian invasion of Ukraine. Existing literature on the military conflict in Ukraine predominantly addressed the destruction of hospital infrastructure, security risks, restricted mobility, disrupted supply chains, power outages, and medical personnel shortages^9–12^. Only one study, involving data from 10 Ukrainian hospitals, reported an increase, not statistically significant, in the percentage of low birth-weight newborns after the invasion^12^. However, that study did not provide specific data on birth-weights or due dates, and its comparison of pre- and post-invasion periods has a potential sampling bias, as it included births before and soon after the outbreak of war.

The rate of low birth-weight among our study participants, both before the war (1.2%) and after (2.4%) the war is much lower than the rate for Ukraine in 2015–2020 (5.7%), based on data collected by UNICEF^13^. This is not due to the study sample size since the 95% confidence intervals for the rates are 0.3–3.1% and 1.1–4.5%, respectively. The Kyiv location of the Eastern-Ukrainian Center for Medical Genetics and Prenatal Diagnosis may have contributed, since low birth-weight tends to be more common in rural compared with urban areas. The most likely explanation is the study design which excluded women with an intrinsic chance of a high risk pregnancy, mainly twins, maternal conditions and previous obstetric history.

The rate of low birth-weight in the current study doubled after the Russian invasion but the difference was not statistically significant. Although the study had sufficient statistical power to detect a difference in median birth-weight it was under-powered for comparing rates of low birth-weight. Nonetheless, UNICEF data show generally downward trends in the rate of low birth-weight from 2000 to 2020^13^. Against this background the upward trend reported in the current study is noteworthy and an important topic for future research.

While all available studies worldwide confirm the impact of military conflict on birth-weight, they also highlight variability based on socioeconomic conditions. The adverse effects are more pronounced in settings with lower education and income levels, or where disruptions to healthcare services and food supply chains are severe^1,3,14,15^.

The literature reveals conflicting findings regarding the influence of gestational age on birth-weight. Some studies suggest that the first trimester is the most critical period for stress exposure, leading to significant weight reductions^3,15^. Others argue that the second and third trimesters carry a greater risk^14^. In contrast, some researchers report that stress in the first and second trimesters result in significant weight reduction, while exposure during the third trimester has no impact^6^. One study found the same effect and no correlation between the timing of prenatal exposure and birth-weight^16^.

Among widely discussed causes of lower birth-weights during military conflicts are severely overburdened health systems, poor maternal and newborn care standards, exacerbated by displacement, restricted healthcare access, insufficient medical supplies, food insecurity, and traumatic stress.

Our study focused on women receiving prenatal care at the Kyiv branch of the Eastern Ukrainian Center for Medical Genetics and Prenatal Diagnostics, before and after the onset of the full-scale invasion. There were no restrictions or disruptions in access to healthcare and food supplies or problems with the provision of prenatal screening tests. Biochemical screening tests and ultrasound examinations were performed using the same approach, equipment, and personnel throughout the study period. Hence, post-traumatic stress likely played a key role, as many women endured anxiety, depression, sheltering during bombardments, and suffering the death of family members and friends.

The negative impact of prenatal stress on fetal development is well documented, including in animal studies. Literature reviews^17,18^ consistently show that the most robust, consistent, and well-replicated result is the association between prenatal stress and low birth-weight. An independent association of military conflict-induced post-traumatic stress with reduced birth-weight was shown in Colombia^15^, in a war-affected area of Pakistan^19^, and in Belgrade after the 1999 bombing of Serbia^16^. In the Belgrade cohort, mothers exposed to stress gave birth to infants with an average weight reduction of 86 g compared to controls (*P* < 0.001) when controlling for the confounding effects of infant length, and there was no significant correlation between the trimester of stress exposure and birth-weight.

Addressing neonatal weight loss in conflict contexts poses tactical, clinical, and strategic challenges requiring immediate action from healthcare providers, humanitarian organizations, and policymakers. Understanding this issue is crucial for several reasons. Firstly, it highlights the immediate need for humanitarian aid targeted specifically towards maternal and newborn health during conflicts. Secondly, it underscores the importance of developing policies that protect and prioritize neonatal health as a fundamental aspect of crisis response and recovery efforts. Finally, research into neonatal weight loss in these settings can inform more effective health protocols and preparedness strategies to mitigate infant morbidity and mortality in future conflicts.

## Data Availability

The research data supporting this publication are available on request from the University of Exeter’s institutional repository at: https://doi.org/10.24378/exe.5586.
